# Persistence of Nocturnality in Decapitated and Bisected Flatworms

**DOI:** 10.1177/07487304231158947

**Published:** 2023-03-24

**Authors:** Shauni E. T. Omond, John A. Lesku

**Affiliations:** School of Agriculture, Biomedicine and Environment, La Trobe University, Melbourne, VIC, Australia

**Keywords:** blastema, brain, *Girardia*, neoblast, planarian

## Abstract

The ability of flatworms to regenerate entire brain structures, and indeed much of their body from mere fragments of the whole animal, presents the unique opportunity to observe the development of day-night rhythms in adult animals. In many animals, young are arrhythmic, and their species-specific timing of activity develops as the animal matures. In this study, we created two flatworm cohorts, housed in isolation, that were regenerating either (1) the brain in a decapitated animal, or (2) major body structures in a bisected, tailless animal. In this way, we observed how bisection influenced the level of activity and diel rhythmicity, and how these developed as each flatworm regenerated. Here, we demonstrate that intact flatworms were predominantly active at night, with peaks in activity seen in the hours after lights-off and before lights-on. While decapitated and tailless flatworms could still move, both were less active than the original animal, and both segments retained a nocturnal lifestyle. Furthermore, decapitated flatworms, once regenerated, again showed a U-shaped pattern of nocturnal activity reminiscent of the two night-time peaks seen in the original animal. These results could be used to further investigate how regeneration may affect motor control and motor output, or to further investigate the presence of a clock in the flatworm brain.

Physiology and behavior are often aligned with the time-of-day via a circadian clock. The function of this clock is to synchronize organismal biology with predictable changes in the environment (Peschel and Helfrich-Forster, 2011). Animals can be categorized into one of four different types depending on their phasing of activity. Some are active during the day (diurnal) or night (nocturnal) or are active at dawn and dusk (crepuscular); others have no obvious alignment with the 24-h day (cathemeral) (Ball, 1992). Diverse examples of behavioral diel rhythmicity include the daily timing of emergence in night-active bats to hunt night-active insects (Acharya et al., 2015; Murugavel et al., 2021), singing in songbirds to deter rivals and attract mates (Moran et al., 2019), and development in insects (Eban-Rothschild et al., 2012; Bloch et al., 2013). By looking at the activity rhythms of animals, we can begin to understand the adaptive significance of patterns that shape species’ behavior (Steiger et al., 2013; Klein et al., 2014; Kelly et al., 2020).

Regulation of the timing of activity develops over early ontogeny. Young animals are typically more arrhythmic than adults, and their diel pattern is entrained, over time, by light-dark cycles and, in some cases, the behavioral pattern of conspecifics (Jilge, 1993; Steiger et al., 2013; Yadav et al., 2014; Yerga et al., 2015). Human babies are cathemeral up to (around) 3 months of age, and become diurnal thereafter (Rivkees, 2003). Similarly, juvenile honeybees (*Apis mellifera*) take 2 weeks to exhibit a day-active rhythm that resembles that of adults (Eban-Rothschild et al., 2012; Klein et al., 2014).

An interesting, yet untapped, system for studying the development of day-night rhythms comes from platyhelminth flatworms. Flatworms are simple invertebrates. Nocturnal by nature, their rhythm persists even in the absence of photoperiodic cues, suggestive of an endogenous circadian clock (Omond et al., 2017). Even more interestingly, despite being closely related to other lophotrochozoans, that is, annelid (segmented) worms and mollusks (Tessmar-Raible, 2003), flatworms have secondarily lost their circulatory and respiratory systems, and endocrine glands. What remains is (1) a centralized nervous system, complete with bilobed brain (Roberts-Galbraith and Newmark, 2015); (2) a need for sleep that is regulated by sleep-wake history and induced by the evolutionarily conserved neurotransmitter gamma-aminobutyric acid, or GABA (Omond et al., 2017; Omond et al., 2022), (3) two cephalic eyespots (or ocelli) for detecting variability in light intensity; and (4) a digestive system in which food is ingested through the pharynx located near the mid-point of their anterior-posterior axis. Importantly, flatworms also have a unique and wholly remarkable ability to regenerate all body tissues from mere slivers of the entire animal via stem cells called neoblasts (Adler et al., 2014). Once injured, neoblasts migrate to the wound, and differentiate and proliferate into the missing cell types. This even includes the de novo regeneration of the brain (Umesono and Agata, 2009). Perhaps not surprisingly, flatworms are commonly used as a model for neurodegenerative diseases (Sandmann et al., 2011; Rink, 2018; Ivankovic et al., 2019; Goldman and Poss, 2020). The ability of bisected flatworms to regenerate affords the opportunity to observe the behavioral patterns associated with day-night rhythms and their implications during this peculiar take on development.

## Methods

Free-living flatworms (*Girardia tigrina*) were wild-caught by Southern Biological (Melbourne, Australia) and housed at nearby La Trobe University. The flatworms were maintained on a 12:12 light: dark (LD) photoperiod with lights-off at 2000 h, and housed in a temperature controlled room at 19 ± 1 °C; this temperature is 5 °C warmer than in Omond et al. (2017) following other studies on regeneration (Hammoudi et al., 2018; Ding et al., 2019). Upon arrival, the flatworms were group housed in 15-cm diameter ceramic bowls (50 flatworms per bowl) filled with clean spring water; flatworms were provided hard-boiled egg yolk for 2 h twice weekly. After 14 days, and for the duration of the study, animals were moved to individual ceramic bowls (7-cm diameter), filled with spring water and fed prior to the start of each of three video recording sessions.

Animal behavior was filmed using a custom-built system that consisted of two aluminum “WormBox” frames (805 × 600 × 600 mm^3^ high). Within each frame, a single camera (A602f; Basler AG, Ahrensburg, Germany) was positioned above the testing arena. The camera mount had four radiating arms, with each arm terminating at infrared (IR) LEDs (950 nm). The IR lights provided constant illumination for the camera to ensure that there was no difference in image quality between the day and night; flatworms do not respond to IR light (Paskin et al., 2014). For the benefit of the flatworms, the two side walls were lined with dim, white LEDs (64 ± 2 lux; Suppl. Fig. S1) to provide daylight mimicking the low light intensity reminiscent of their turbid freshwater habitat.

**Figure 1.**
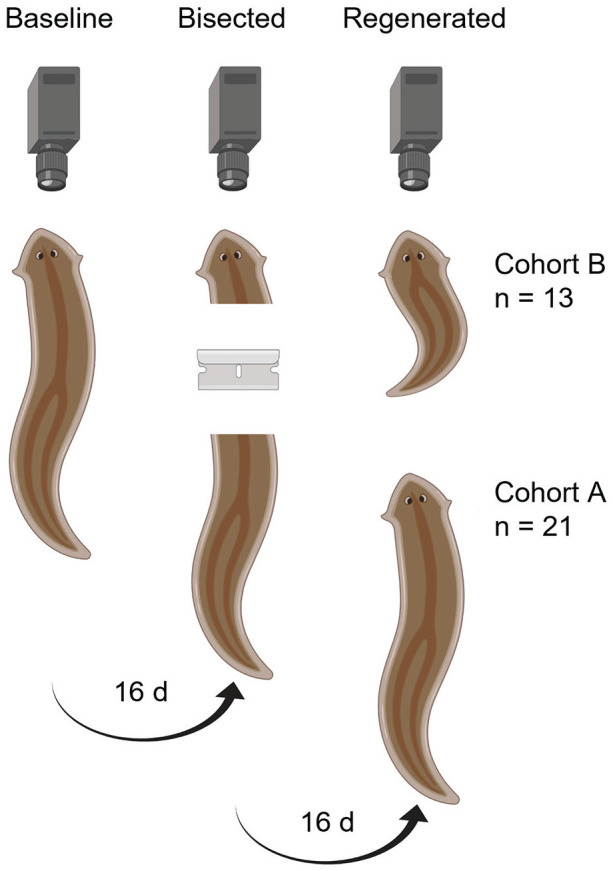
Regeneration protocol. The original animal was fed and then video recorded for 55 h (*baseline*). After 15 to 16 days, the flatworm was fed, decapitated, and filmed another 55 h (*bisected*). A third recording took place 15 to 16 days later, in which the animal was again offered food and recorded for 55 h (*regenerated*). Cohort A refers to the decapitated flatworm and subsequently regenerated head; Cohort B relates to the tailless head and regenerated tail. Created using BioRender.com.

At the start of the experiment, flatworms were offered hard-boiled egg yolk from 1000 to 1200 h, placed into the WormBox at 1300 h, and recorded for 55 h (Figure 1). Two mL of spring water was gently added to each bowl at the onset of Night 2 to replenish water lost from evaporation. On the day of their next recording (15-16 days after the conclusion of the baseline session), the animals were fed (as above) and cut into anterior and posterior segments using a razor blade. The cut was made posterior to the eyespots to remove the head. Flatworms feed using their pharynx which is located near the mid-point of their body. Consequently, the headless tail (Cohort A; *n* = 21) retained the pharynx, whereas the tailless head (Cohort B; *n* = 13) did not. (Sample sizes differed between the two cohorts as not all animals regenerate their other half in the time allotted). Both segments were placed into separate dishes at 1300 h and recorded for another 55 h. As above, animals were not fed until the day of the third recording, 15 to 16 days later. After feeding, the flatworms were again recorded for 55 h. Note, approximately 10 days post-bisection, eyespots and head shape had regenerated to the original appearance.

The WormBox recording system took a photograph at the top of every minute and compiled these images into 55-h videos. The first 7 h of each 55-h video was excluded such that analyses commenced at lights-off; the videos were then manually scored to determine whether the flatworm had changed position during the intervening minute. Movements, and the absence thereof, were scored as active and restful, respectively. Data were then converted to a percentage of each hour spent active. These data were summarized as 12-h night- and 12-h day-time means (i.e., the average of the two 12-h nights and two 12-h days per flatworm). Data were also visualized by 3-h (quarterly) time bins to reveal finer-scale temporal patterns. For the 12-h data, a repeat-measures analysis of variance (ANOVA) determined whether decapitation influenced activity levels across the night and day, followed by paired *t* tests to determine which contrasts reached significance for the headless tail (Cohort A) and tailless head (Cohort B). For the 3-h bins, data could not be normalized such that we had to use a (non-parametric) Friedman’s Test, and Dunn’s multiple comparison test to determine the level(s) at which significance was reached for Cohorts A and B. Outliers were removed from the quarterly time bins using Grubb’s iterative statistics. Statistical analyses were conducted using GraphPad Prism 9.0 (GraphPad Software, La Jolla, USA).

## Results

We begin by summarizing the gross effect of decapitation and regeneration on activity during the night and day for Cohort A (*n* = 21) and Cohort B (*n* = 13).

### Cohort A

**Figure 2.**
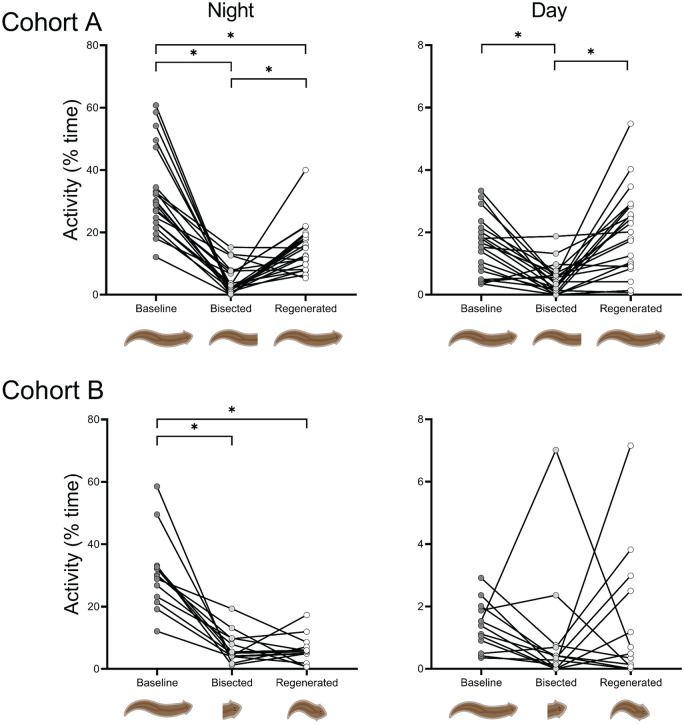
The amount of activity per flatworm for the timepoints: *baseline, bisected*, and *regenerated*. Comparison of night-time (*left*) and day-time (*right*) mean activity over two 12-h periods for Cohorts A (*top*) and B (*bottom*). Significant pairwise comparisons are marked by asterisks.

Under baseline conditions (intact), flatworms are nocturnal, spending 32.7% and 2.0% of the night and day moving, respectively (Figure 2). The amount of activity was influenced by decapitation at night (*F*_2,40_ = 59.88, *p* < 0.001) and during the day (*F*_2,40_ = 17.14, *p* < 0.001). Specifically, once decapitated, the animals still moved, but did so far less during the night (paired *t* test: *t*_20_ = –10.44, *p* < 0.001) and day (*t*_20_ = –4.86, *p* < 0.001). After regenerating a new head, flatworms were more active than their decapitated tails at night (*t*_20_ = 5.55, *p* < 0.001), but remained less active than their original head (*t*_20_ = –5.69, *p* < 0.001). Conversely, during the day-time, the regenerated flatworms were more active than the headless tails (*t*_20_ = 5.08, *p* < 0.001), and as active as the original animal (*t*_20_ = 1.75, *p* = 0.09). Interestingly, decapitated flatworms maintained their nocturnal lifestyle in that the headless tails were still more active at night than during the day (*t*_20_ = 4.51, *p* < 0.001).

### Cohort B

The amount of activity observed in Cohort B was influenced by bisection during the night (*F*_2,24_ = 37.66, *p* < 0.001), but not in the day (χ^2^ = 4.27, *p* = 0.12) (Figure 2). The lack of significance in the latter should be viewed with caution given the outlier in the bisected group, and a smaller sample size for Cohort B. The tailless heads and regenerated flatworms were both less active than the original flatworms at night (*t*_12_ = –6.78, *p* < 0.001; *t*_12_ = –7.92, *p* < 0.001, respectively), yet the regenerated animals were as restful as the tailless heads (*t*_12_ = 0.015, *p* = 0.98). Similar to Cohort A, the tailless heads still showed a nocturnal rhythm by being more active at night than during the day (*W*_12_ = 91, *p* < 0.001).

Next, we explore the finer-scale temporal dynamics of these effects by looking at the data in 3-h (quarterly) time bins for both cohorts. While doing so, we were mindful of the increased risk of a Type I error and adjusted the per-comparison alpha, for Dunn’s multiple comparison tests, based on the Bonferroni method (α = 0.0016) to maintain the family-wise error rate at 0.05.

### Cohort A

**Figure 3.**
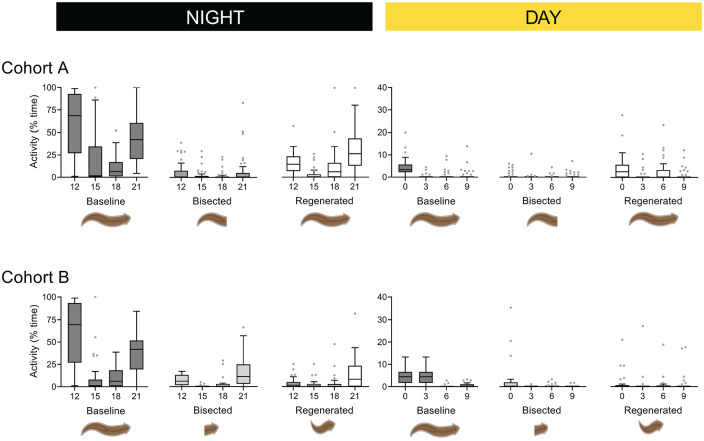
The amount of activity shown as quarterly boxplots for *baseline, bisected*, and *regenerated* flatworms of Cohort A (*top*) and Cohort B (*bottom*) during the night (*left*) and day (*right*). Time-of-day on the x-axis is denoted as *zeitgeber* time at the start of each 3-h time bin. The top and bottom edge of each box reflect first and third quartiles, respectively; the interior line denotes the median; and whiskers show minimum and maximum values; data points are statistical outliers not identified by Grubb’s iterative test.

Under baseline conditions at night, flatworms exhibit a U-shaped activity pattern (χ^2^ = 63.08, *p* < 0.001; Figure 3). That is, they are most active in the 3 h after lights-off, and 3 h before lights-on. The decapitated tails lost this bimodal rhythm (χ^2^ = 5.13, *p* = 0.16) and were less active during zeitgeber times 12, 18, and 21 of the night, and the first quarter of the day (Table 1). Limited day-time significance is not unexpected, given that nocturnal flatworms are rarely active during the day. The regenerated flatworms regained the U-shaped activity pattern (χ^2^ = 54.71, *p* < 0.001), even while they remained less active during the first quarter of the night.

**Table 1.** Details of statistical significance for Cohort A (*top*) and Cohort B (*bottom*) in Figure 3 across days (D) and nights (N); α = 0.0016.

Night (N) / Day (D)	Zeitgeber Time	Baseline vs. Bisected		Baseline vs. Regenerated	
		*z*	*p*	*z*	*p*
N	12	6.17	<0.001	3.98	<0.001
N	15	2.78	0.01	2.95	0.006
N	18	4.42	<0.001	0.62	>0.99
N	21	5.51	<0.001	0.87	0.76
D	0	4.91	<0.001	1.96	0.09
D	3	0.05	>0.99	0.38	>0.99
D	6	0.44	>0.99	1.42	0.31
D	9	0.22	>0.99	0.54	>0.99
N	12	4.16	<0.001	5.41	<0.001
N	15	2.50	0.02	0.42	>0.99
N	18	2.63	0.01	2.98	0.005
N	21	2.43	0.03	2.98	0.005
D	0	2.56	0.02	3.26	0.002
D	3	4.99	<0.001	4.58	<0.001
D	6	0.76	0.89	0.90	0.73
D	9	1.11	0.53	0.14	>0.99

Zeitgeber time denotes the start of each 3-h time bin.

### Cohort B

As with Cohort A, the activity rhythm of flatworms is nocturnal, with very little activity during the day and a U-shaped pattern of activity at night (χ^2^ = 43.49, *p* < 0.001; Figure 3). Interestingly, the tailless heads and regenerated animals retained this bimodal rhythm at night (χ^2^ = 41.97, *p* < 0.001; χ^2^ = 10.97, *p* = 0.012; respectively). Both the tailless heads and regenerated flatworms were less active than the original animals during the first quarter of the night and second quarter of the day (Table 1). As with the headless tails, here, the tailless heads retained a nocturnal lifestyle. During the day, the activity of the tailless heads, and regenerated flatworms, were homogeneously low, not so dissimilar to that of the original animals.

## Discussion

Nocturnal flatworms that have lost their head maintained a night-active lifestyle, even while finer-scale patterns were disrupted. This was also the case for heads that parted with their tails. To our knowledge, this is the first demonstration that both parts of a bisected flatworm have an activity rhythm. Planarians detect, and avoid, light not only using their eyespots (or ocelli), but also via extraocular photoreceptors diffusely located throughout their bodies (Shettigar et al., 2017, 2021). In this way, both the headless tail and tailless head were equally able to detect light to maintain a nocturnal pattern of activity. That said, day-night rhythmicity endures in flatworms even in the absence of photoperiodic cues (Omond et al., 2017). Taken together, the persistence of nocturnality could be due to an endogenous circadian clock, entrained by extraocular photoreceptors (Cohort A) and ocelli (Cohort B).

Once bisected, flatworms are more quiescent, particularly at night. Studies have looked at regeneration from cellular and genetic perspectives (Reddien and Sánchez Alvarado, 2004; Goldman and Poss, 2020; Jin et al., 2022). Once injured, a flatworm decreases the surface area of the wound site by contracting the muscles along the wound’s edge; secreted mucous acts as a protective barrier. Totipotent cell migration and proliferation occur along the wound site to form a blastema (or epithelial bud) (Reddien and Sánchez Alvarado, 2004; Saló, 2006; Roberts-Galbraith and Newmark, 2015). Tissue polarity and pre-existing tissue along the wound edge triggers the regeneration of the missing tissues, such that a decapitated fragment would regenerate de novo a new head. The time taken to regenerate tissue depends on the amount of tissue missing (Reddien and Sánchez Alvarado, 2004). The chronic reduction of activity we observed is likely due to the reallocation of finite energy stores toward regenerative processes, rather than an inability to move since bisected flatworms were still active, albeit at lower levels.

Although we do not know whether restful regenerating flatworms were asleep, or immobile but awake, the finding of reduced activity after bisection might provide intriguing (albeit speculative) insight into the function of sleep. First, young animals sleep more than adults. This basic developmental pattern has been observed in mammals (Jouvet-Mounier et al., 1969; McGraw et al., 1999), birds (Scriba et al., 2014), and invertebrates (Greenspan et al., 2001). As a result, sleep is thought to play a role in the maturation of the central nervous system (Roffwarg et al., 1966), and has at least the potential to do so in decapitated flatworms (Omond et al., 2017). Second, sleep saves energy by (1) lowering metabolic rate, (2) not doing something more energetically demanding, and (3) partitioning metabolic processes to either sleep or wakefulness (Lesku et al., 2006; Schmidt, 2014; Ferretti et al., 2019; Kelly et al., 2022; Lesku and Schmidt, 2022). The energy saved by sleeping can then be reallocated to other purposes, such as investment into the immune system (Preston et al., 2009). A non-exclusive idea posits that increased restfulness could be a means to reduce predatory encounters (Siegel, 2009). In any case, restful flatworms could be asleep to allow sleep-dependent processes to facilitate regeneration of the other one-half of the animal. To determine whether this idea has merit would require a method for characterizing sleep that does not rely on behavioral responses (Omond et al., 2017, 2022), possibly related to calcium imaging (Bushey et al., 2015; Cox et al., 2016), immunofluorescence (Nishimura et al., 2007), optogenetic techniques (Turek et al., 2013), or respirometry (Lemieux and Warren, 2012; Osuma et al., 2018).

A head, or perhaps specifically a brain, could be important for the fine-scale organization of night-time activity. The headless tail loses the U-shaped pattern of activity seen in the original animal, which is then regained in the regenerated flatworm (Cohort A). After decapitation, the reduction of activity could have arisen from impaired motor control or output, as the tailless head retains the U-shaped pattern (Cohort B). Several lines of evidence point toward a circadian clock in flatworms. (1) Flatworms are more active at night, (2) a rhythm that persists in the absence of photoperiodic cues; (3) with anticipatory behavior occurring prior to photoperiodic changes (Omond et al., 2017); (4) flatworms show diel variation in the production of melatonin, serotonin, and arylalkylamine *N*-acetyltransferase under LD and DD conditions (Itoh et al., 1999; Itoh and Igarashi, 2000). (5) Molecular candidates for a time-keeper in flatworms could relate to expression of the gene *Smed-Tim*, a homolog of the mammalian *Tim* (Tsoumtsa et al., 2017); genes that regulate circadian processes in other animals, including sponges, cnidarians, and fruit flies (e.g., *Clock, per*) are absent in planarians (Reitzel et al., 2010). Ultimately, an understanding of the exact machinery of the circadian clock in flatworms remains unknown (Hinrichsen et al., 2019). Recording flatworms under constant dark conditions would provide insight into whether the bimodal pattern of night-time activity originates from clock regulation.

The unique ability of flatworms to regenerate a whole animal from very small fragments of itself constitutes a novel system for understanding many biological processes. Planarians are promising in research related to drug and alcohol interactions (Ramakrishnan and Desaer, 2011; Moustakas et al., 2015), locomotory behaviors (Deochand et al., 2018), circadian rhythms (Gutiérrez-Gutiérrez et al., 2017; Tsoumtsa et al., 2017), cognition (Abbott and Wong, 2008; Shomrat and Levin, 2013; Neuhof et al., 2016; Deochand et al., 2018), and sleep (Omond et al., 2017; Omond et al., 2022), all within the same genetic individual. We offer our results as a starting point for future investigations into how regeneration may affect motor control and motor output, and the presence of a clock in the flatworm brain.

## Supplemental Material

sj-docx-1-jbr-10.1177_07487304231158947 – Supplemental material for Persistence of Nocturnality in Decapitated and Bisected Flatworms Click here for additional data file. Supplemental material, sj-docx-1-jbr-10.1177_07487304231158947 for Persistence of Nocturnality in Decapitated and Bisected Flatworms by Shauni E. T. Omond1 and John A. Lesku2 in Journal of Biological Rhythms
