# Supplementary material for: Persistence of Nocturnality in Decapitated and Bisected Flatworms
Source: J Biol Rhythms. 2023 Mar 24;38(3):269–77. doi: 10.1177/07487304231158947 (PMC10278384; doi:10.1177/07487304231158947)
Supplement: sj-docx-1-jbr-10.1177_07487304231158947 – Supplemental material for Persistence of Nocturnality in Decapitated and Bisected Flatworms [file sj-docx-1-jbr-10.1177_07487304231158947.docx]

**SUPPLEMENTARY MATERIAL**


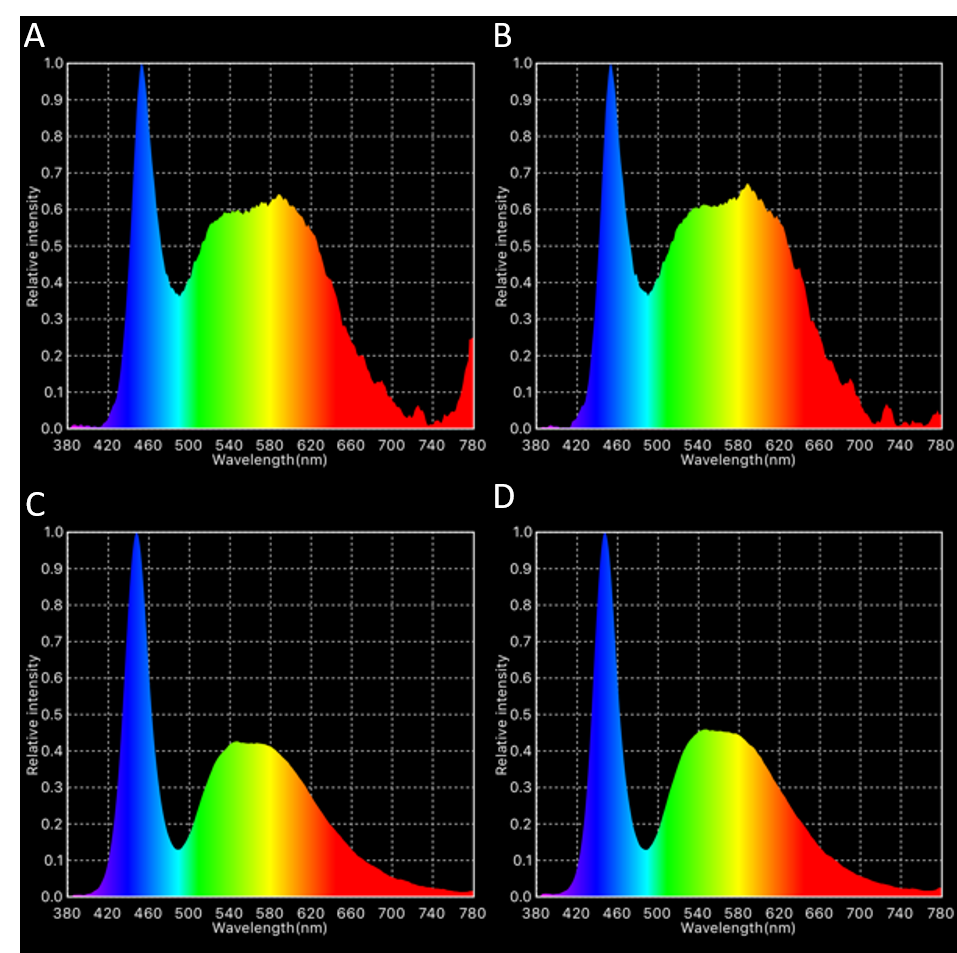


Figure S1. Spectra of the dim (64 ± 2 lux) light sources used to illuminate the flatworms during the light phase of the experimental recordings (A-B) and under maintenance conditions (C-D).
